# How Often Do They Have Sex? A Comparative Analysis of the Population Structure of Seven Eukaryotic Microbial Pathogens

**DOI:** 10.1371/journal.pone.0103131

**Published:** 2014-07-23

**Authors:** Nicolás Tomasini, Juan José Lauthier, Francisco José Ayala, Michel Tibayrenc, Patricio Diosque

**Affiliations:** 1 Unidad de Epidemiología Molecular (UEM), Instituto de Patología Experimental, Universidad Nacional de Salta-CONICET, Salta, Salta, Argentina; 2 Department of Ecology and Evolutionary Biology, University of California Irvine, Irvine, California, United States of America; 3 Maladies Infectieuses et Vecteurs Ecologie, Génétique, Evolution et Contrôle, MIVEGEC (IRD 224-CNRS 5290-UM1-UM2), IRD Center, Montpellier, France; St. Petersburg Pasteur Institute, Russian Federation

## Abstract

The model of predominant clonal evolution (PCE) proposed for micropathogens does not state that genetic exchange is totally absent, but rather, that it is too rare to break the prevalent PCE pattern. However, the actual impact of this “residual” genetic exchange should be evaluated. Multilocus Sequence Typing (MLST) is an excellent tool to explore the problem. Here, we compared online available MLST datasets for seven eukaryotic microbial pathogens: *Trypanosoma cruzi*, the *Fusarium solani* complex, *Aspergillus fumigatus*, *Blastocystis* subtype 3, the *Leishmania donovani* complex, *Candida albicans* and *Candida glabrata*. We first analyzed phylogenetic relationships among genotypes within each dataset. Then, we examined different measures of branch support and incongruence among loci as signs of genetic structure and levels of past recombination. The analyses allow us to identify three types of genetic structure. The first was characterized by trees with well-supported branches and low levels of incongruence suggesting well-structured populations and PCE. This was the case for the *T. cruzi* and *F. solani* datasets. The second genetic structure, represented by *Blastocystis* spp., *A. fumigatus* and the *L. donovani* complex datasets, showed trees with weakly-supported branches but low levels of incongruence among loci, whereby genetic structuration was not clearly defined by MLST. Finally, trees showing weakly-supported branches and high levels of incongruence among loci were observed for *Candida* species, suggesting that genetic exchange has a higher evolutionary impact in these mainly clonal yeast species. Furthermore, simulations showed that MLST may fail to show right clustering in population datasets even in the absence of genetic exchange. In conclusion, these results make it possible to infer variable impacts of genetic exchange in populations of predominantly clonal micro-pathogens. Moreover, our results reveal different problems of MLST to determine the genetic structure in these organisms that should be considered.

## Introduction

The Predominant Clonal Evolution (PCE) model [1]–[5] deals with pathogen population structure rather than with the precise cytological mode of reproduction. According to the PCE model, clonality is defined as severely restrained genetic recombination, a definition that is accepted by many, if not most, authors working on microbial pathogens (viruses, bacteria, parasitic protozoa and fungi) [3]. The two main consequences of PCE are a strong linkage disequilibrium (LD), or nonrandom association of genotypes at different loci, and a structuration of pathogen populations into stable, discrete genetic clusters, or “near-clades”. This term has been coined [3], because the term “clade” is improper for the present purpose, since residual genetic recombination always goes on in pathogen populations. The model has been challenged by recent studies showing limited recombination in some populations of *Trypanosoma cruzi* [6] and by distinguishing self-fertilization and inbreeding from “strict” clonality [7]. However, limited recombination in particular cycles and selfing/inbreeding are clearly included in the PCE model [3], [4].

It is clear that some recombination occurs or has occurred in pathogenic microeukaryotes. Ancient, strict clonal lineages seem to be rare in nature [8]. However, classical population genetics tools designed for higher sexual organisms present several problems when micropathogens are concerned [3], [4]. Unconventional sexuality, genome-wide mitotic gene conversion and aneuploidy are examples. Particularly, many of these tests are based on the working hypothesis of diploidy [9], while widespread aneuploidy seems to be a common feature in parasites such as *Leishmania* [10] and fungi [11]. Phylogenetic analyses implementing multiple genes are an interesting alternative to analyze this problem on a longer time-scale. As a matter of fact, one important (but not the only) cause of incongruence among gene trees is genetic exchange. Paralogy in gene families, very different evolutionary rates among fragments and different selective pressures are other causes of incongruence.

Multilocus Sequence Typing (MLST) [12], a widely used typing method, has the advantage of a fair resolution power to analyze genetic diversity at population levels. The methodology relies on sequencing fragments of several housekeeping genes (generally five to seven fragments). Since single copy fragments from housekeeping genes are preferred for MLST, the possibility of paralogy or very different evolutionary rates is reduced. Consequently, genetic exchange is the major cause of incongruence in MLST data. Different multilocus genotypes (MLG) can be defined by MLST and they are called “sequence types” (STs). Datasets of many species are available [13], [14]; however, the number of studies dealing with comparisons of MLST data among species is limited [15].

Most studies in eukaryotic micropathogens define trees and clusters based on classical phylogenetic methods. These trees are based on the concatenation of the sequences of different loci. This approach is susceptible to biases caused by recombination and incongruence among loci. These biases are not considered in most papers dealing with MLST in eukaryotic micropathogens.

In the present study, we first analyze the genetic structure in MLST datasets of seven eukaryotic microbial pathogens: *Trypanosoma cruzi*, the *Fusarium solani* species complex, *Aspergillus fumigatus*, *Blastocystis* subtype 3, the *Leishmania donovani* complex, *Candida albicans* and *Candida glabrata*. Then, we propose classification criteria of MLST datasets according to the degree of genetic structuring and the impact of genetic exchange in the populations under survey. Then, we evaluate the efficiency of MLST in the different datasets to determine clusters. Lastly, we propose criteria to define clusters or “near-clades” [3] based on MLST data.

## Materials and Methods

### 2.1 Datasets

The *Candida glabrata* dataset (Cg) using the MLST scheme proposed by Dodgson et al. [16] and the *Candida albicans* dataset (Ca) using the MLST scheme proposed by Bougnoux et al. [17] were downloaded from MLST.net [13] (http://cglabrata.mlst.net/and http://calbicans.mlst.net respectively). In addition, a search in the available genomes of *C. albicans* showed that the 7 used housekeeping fragments are single-copy. The fragments for *C. glabrata* are also single-copy with the exception of *FKS* gene (1,3-beta-glucan synthase). This gene has one paralogous; however, this paralogous gene has low pairwise identity (78%) and it has not annealing sites for the used primers. The *Aspergillus fumigatus* database (Af) using the MLST scheme proposed by Bain et al. [18] and the *Blastocystis* ST3 dataset (B3) using the scheme proposed by Stensvold et al. [19] were downloaded from the pubmlst site [14] (http://pubmlst.org/afumigatus/and http://pubmlst.org/blastocystis/). Six out of seven gene fragments of *A. fumigatus* were single-copy housekeeping genes according the BLAST search. Just one fragment (*SODB*, superoxide dismutase) had a paralogous copy but with low pairwise identity (69.2%) and without annealing sites for used primers. All fragments used for typing *Blastocystis* ST3 dataset were single-copy regions of the mitochondrion-like genome and there were no paralogous copies in the nuclear genome. It is important to note that those regions used in *Blastocysti*s ST3 were non-coding regions. Sequences for the *Leishmania donovani* complex (Ld) [20], *Trypanosoma cruzi* (Tc) [21] *and Fusarium solani* complex (Fs) [22] were downloaded from GenBank using published accession numbers. Additional sequences for *T. cruzi* are available at Genbank under KF889442–KF889571 accession numbers. The MLST scheme used for *T. cruzi* proposed by Lauthier et al. [21] is the best combination of 7 loci concerning this parasite. Five out of these seven housekeeping loci were single copy. The Small GTP-binding protein Rab7 (*GTP)* had paralogous in BLAST search for CL Brener strain genome but with low pairwise identity (<75%, without annealing site for the used primers). In addition, Just Superoxide dismutase (*SODB*) had two copies with high pairwise identity (99%) and both with annealing sites for the used primers. All housekeeping gene fragments used for *F. solani* species complex and *Leishmania donovani* complex were single-copy. Only one strain for each sequence type (ST) was used in the analysis unless otherwise specified. Seq-Gen v1.3.2 [23] was used to simulate a dataset of six fragments under the full congruence hypothesis (Datasets here referred to as CONG). Full congruence means that all fragments in the dataset have evolved over the same phylogenetic tree. Because genetic exchange is a cause of incongruence, simulated datasets obtained under the full congruence hypothesis may be considered as datasets having evolved under the expectations of strict clonality. The CONG datasets were simulated under a tree of concatenated fragments for *C. glabrata*. The length of the simulated alignments was set to 500 nucleotides. The number of fragments per dataset was set to 6. The evolutionary model was set to Kimura two-parameters with a transition/transversion ratio of 3 and a proportion of invariable sites of 0.66. The model was selected based on the best model that fit the *T. cruzi* dataset published by Lauthier et al. [21] using jMODELTEST software [24]. In order to compare the datasets against panmictic populations, we simulated four datasets of six fragments each based on the *C. glabrata* tree. Then, labels of taxa were permuted in order to simulate random combinations of alleles as expected under panmictic assumptions. Datasets simulated under panmixia are labeled here with the term RND (from RaNDom associations). For each micropathogen, we also analyzed datasets of 24 STs in order to make comparisons. These 24 STs were randomly selected (selection without reposition). These reduced datasets were made 10 times using different random seeds. Different analyses described below were made for each replica. The number of STs (24 STs) was selected because it is the number of STs for *T. cruzi*, which is the smallest dataset analyzed. In addition, we analyzed reduced datasets of 24 strains (instead of 24 STs) to determine possible bias of using just one representative strain *per* ST. In this case, more than one strain per ST is analyzed in the reduced dataset and the probability of repeated STs depends on the frequency of such STs in the full strain dataset.

### 2.2 Data analysis

Relationships among genotypes were analyzed with MLSTest [25] on concatenated alignments using the Neighbor Joining (NJ) method with uncorrected p-distances considering heterozygous sites as average states. Taking into account that maximum likelihood and Bayesian methods consider heterozygous sites as ambiguous or non-informative, which is undesirable for diploid datasets, these two methods were not used for data analyses. Branch support was calculated by bootstrap with 1,000 replications. In order to make dataset comparisons more simple, those with 40%–60% of branches supported by a bootstrap value higher than 80% were arbitrarily considered as moderately supported, whereas those with more than 60% of branches with a bootstrap value higher than 80% were considered as highly supported.

Additionally, the number of individual fragment trees that support each branch was calculated. We used the term “consensus support” (CS) for this measure, because it is the support that a given branch would have if it appeared in a majority-rule consensus tree. In order to make comparisons, CS was arbitrarily considered moderate for datasets with 30%–60% of branches supported by at least two fragments, and high for datasets with more than 60% of branches supported by at least two fragments. In addition, the mean CS for each dataset was calculated. The mean CS was standardized for datasets with <7 loci. The last measure was calculated dividing mean CS by the number of loci in the dataset and multiplying by 7. In this way, the standardized mean CS is showing the mean CS if the dataset had 7 loci.

Overall incongruence in each dataset was assessed by using the Incongruence Length Difference test relying on the BIO-Neighbor Joining method (ILD-BIONJ) proposed by Zelwer and Daublin [26], with 100 permutations for complete datasets and with 10,000 permutations for reduced datasets. This difference in the number of permutations was set because this test requires much computational time for large datasets. Localized incongruence for each branch in the concatenated tree was evaluated by the number of fragment trees that are topologically incompatible with each considered branch. Topological incongruence (TI) was arbitrarily considered as moderate for datasets of *n* loci having from 20% to 40% of branches with *n*-1 fragments topologically incompatible with the clade in the concatenated tree. Moreover, TI was considered high for datasets with more than 40% of branches with *n*-1 fragments topologically incompatible.

The significance of the localized incongruence was evaluated using the NJ-Localized Incongruence Length difference test [25] as implemented by MLSTest with 1,000 permutations. The Bonferroni correction was applied for multiple comparisons. CS, bootstrap, TI ILD-BIONJ and nj-LILD significance were also analyzed in reduced datasets of 24 STs randomly selected with ten replications in order to make comparisons. This number of STs was selected because is the number of STs for *T. cruzi*, which is the smallest dataset analyzed.

Congruence among distance matrices (CADM) of *C. albicans* datasets and the Mantel test were evaluated using the software CADM [27] with 5,000 random permutations. Evaluation of 4 previously proposed MLST near-clades for *Candida albicans* [28] was made by using the Mantel test with a binary distance matrix in order to model a dataset with only one subdivision (corresponding to the near-clade being tested) as was previously proposed [29]. This matrix is designed by assigning a distance of 1 between STs separated by the branch that define the hypothetical clade and establishing a distance of 0 for all other pairs of STs.

### 2.3 Null hypothesis of different tests and what p values say

Frequently, data (sequences) had inconsistent information suggesting different and incompatible clusterings. Homoplasy (characters shared by two STs that belong to different lineages due to parallelism or reversion rather than to common ancestry) is a cause of contradiction. Another cause of inconsistency is the existence of different evolutionary stories (for example: different evolutionary trees due to genetic exchange, different evolutionary rates, or different selective pressures) of the DNA fragments analyzed. The best tree is generally the one that minimizes the level of inconsistency. ILD-based tests analyze whether the inconsistencies with the concatenated tree are distributed at random among the different fragments (random homoplasy) or whether they are concentrated in certain fragments (incongruence produced by these fragments). Consequently, the null hypothesis of ILD-based tests is the random distribution of homoplasies, or congruence. Incongruence (nonrandom distribution) is the working hypothesis. On the other hand, the null hypothesis of CADM and Mantel tests is random correspondence (lack of correlation) among distance matrices. This means that the null hypothesis (H0) implies full incongruence (strictly, random correspondence) among distance matrices (and consequently trees). The working hypothesis is a statistically significant degree of correlation among distance matrices.

Consequently, a significant p value in the ILD-based test (H0 is rejected) means that at least one fragment produces incongruence. However, a significant p value in a Mantel test means that the hypothesis of full incongruence among distance matrices should be rejected, which means that some level of congruence is recorded among matrices. In this sense, a significant Mantel test (statistically significant correlation) is compatible with a significant ILD-based test (significant incongruence) when there is at the same time some degree of congruence and some degree of incongruence in the dataset. However, as it is the case for any statistical test, a non-significant p value for the Mantel test does not mean that the null hypothesis is corroborated. As a matter of fact, lack of significance could be due to the low power of the test due to insufficient data (statistical type II error).

## Results

### 3.1 Datasets summary

Summarized datasets are presented in Table 1. The number of fragments for each dataset varies from 5 to 7 and the dataset sizes vary from 24 STs for *T. cruzi* to 1,000 STs for *C. albicans*. *A. fumigatus* and *L. donovani* complex showed the lowest numbers of polymorphic sites relative to other datasets. The typing efficiency (Number of ST/number of polymorphisms) was variable among datasets (Table 1). The MLST scheme for *F. solani* complex had the lowest typing efficiency whereas the targets used for *C. albicans* had the highest one, showing 6 STs per polymorphic site.

**Table 1:** Summary of main features of analyzed datasets.

	Datasets^1^						
	Tc	Fs	Af	B3	Ld	Ca	Cg
Number of strains	47	51	98	98	38	1386	212
Number of STs	24	41	28	58	27	1000	68
Number of polymorphysms	125	213	40	181	47	165	125
Number of fragments	7	5	7	5	5	7	6
Typing efficiency^2^	0.2	0.19	0.70	0.32	0.47	6.06	0.54

1 *Tc, Trypanosoma cruzi; Fs, Fusarium solani complex; Af, Aspergillus fumigatus; B3, Blastocystis spp ST3; Ld, Leishmania donovani complex; Ca, Candida albicans; Cg, Candida glabrata.*

2 Typing efficiency: defined as the number of STs per polymorphic site.

### 3.2 Branch support

**Figure 1:**
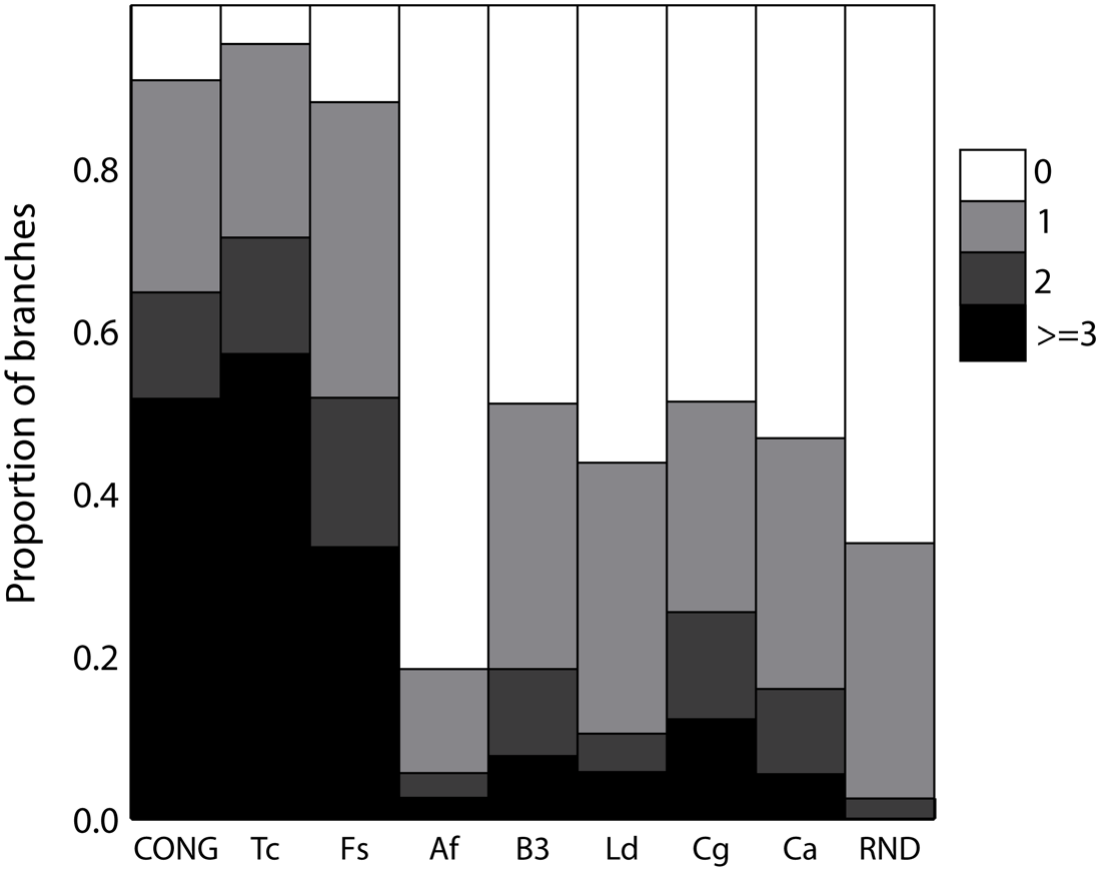
Consensus support distribution for standardized datasets. The color scale-bar represents the level of consensus support that varies from 0 fragment trees (white bars) to ≥3 fragment trees (black bars) supporting the branch in the tree for concatenated alignments. The values are calculated as the mean of 10 replications.

In order to analyze the genetic structure in the datasets, we first evaluated the branch support. It is expected that strongly structured species will have well-supported branches because of low levels of conflict among polymorphisms of different fragments. We first analyzed consensus support (CS). The CS was variable among datasets. The less supported dataset was *C. albicans* and the most supported dataset was *T. cruzi*. For example, we observed that the majority of the branches in *C. albicans* (96.3% of 997 branches) were not supported by any fragment tree (CS = 0, see red branches in Figure S1). On the contrary, 60% of the branches in *T. cruzi* had CS >2 and just 1 (5%) branch was not supported at all. However, both datasets are not directly comparable because they have different number of STs. Because of this, we analyzed random subsamples of 24 STs for each dataset. The *C. albicans* dataset still had low support, since about 53% of the branches were not supported at all and only 5% of the branches were supported by three or more fragments (Figure 1). In addition, the mean CS for *C. albicans* was 0.72 fragments per branch (CI95% = 0.6–0.84). This value –although higher– was relatively close to the observed one for the simulated panmictic dataset (mean CS = 0.42, CI95% = 0.34–0.50). Instead, *T. cruzi* had a mean CS = 3.4 fragments per branch (CI95% = 2.35–4.32). Moreover, the simulated dataset CONG (under strict clonality) was similar to *T. cruzi* with a mean CS = 3.08 (CI95% = 2.81–3.36). *F. solani* complex showed moderate branch support (33% of the branches with CS >2 and a mean CS = 2.7) (Figure 1 and Figure S1). In the other hand, *A. fumigatus*, *L donovani* complex, *Blastocystis spp* and *C. glabrata* showed generally low support values with mean CS ranging from 0.27 to 1.1 fragments per branch (standardized mean CS and corresponding confidence intervals are shown in Figure S2). In addition, similar results were obtained using subsamples of the full set of strains for each micropathogen instead of the set of STs (Figure S3A). This result suggests no bias produced by considering only one representative strain *per* ST.

**Figure 2:**
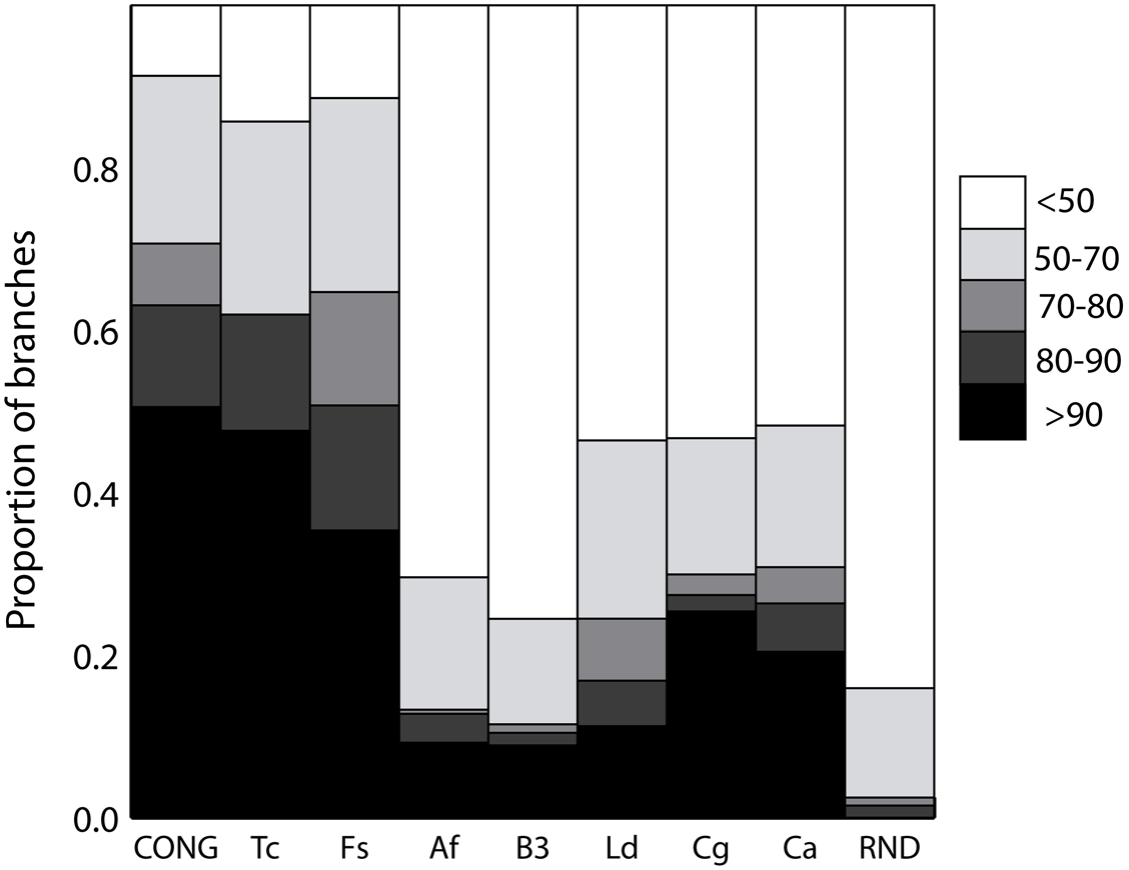
Bootstrap support distribution for standardized datasets. The color scale-bar represents the level of bootstrap support that varies from 0–50% (white bars) to more than 90% (black bars) supporting each branch. The values are calculated as the mean of 10 replications.

We also analyzed bootstrap values for the branches (Figure 2). Bootstrap distribution for each dataset was similar to the CS distribution. Datasets with moderate to high CS (*T. cruzi*, *F. solani* complex and the simulated CONG) had at least 50% of the branches with a bootstrap value higher than 80%. On the other hand, datasets with low CS (*C. albicans*, *C. glabrata*, *L. donovani* complex, *A. fumigatus*, *Blastocystis spp*) had less than 30% of their branches supported by bootstrap values >80%. These results favor the hypothesis of a strong structuration for the *T. cruzi* and *F. solani* complex datasets.

### 3.3 Incongruence

**Figure 3:**
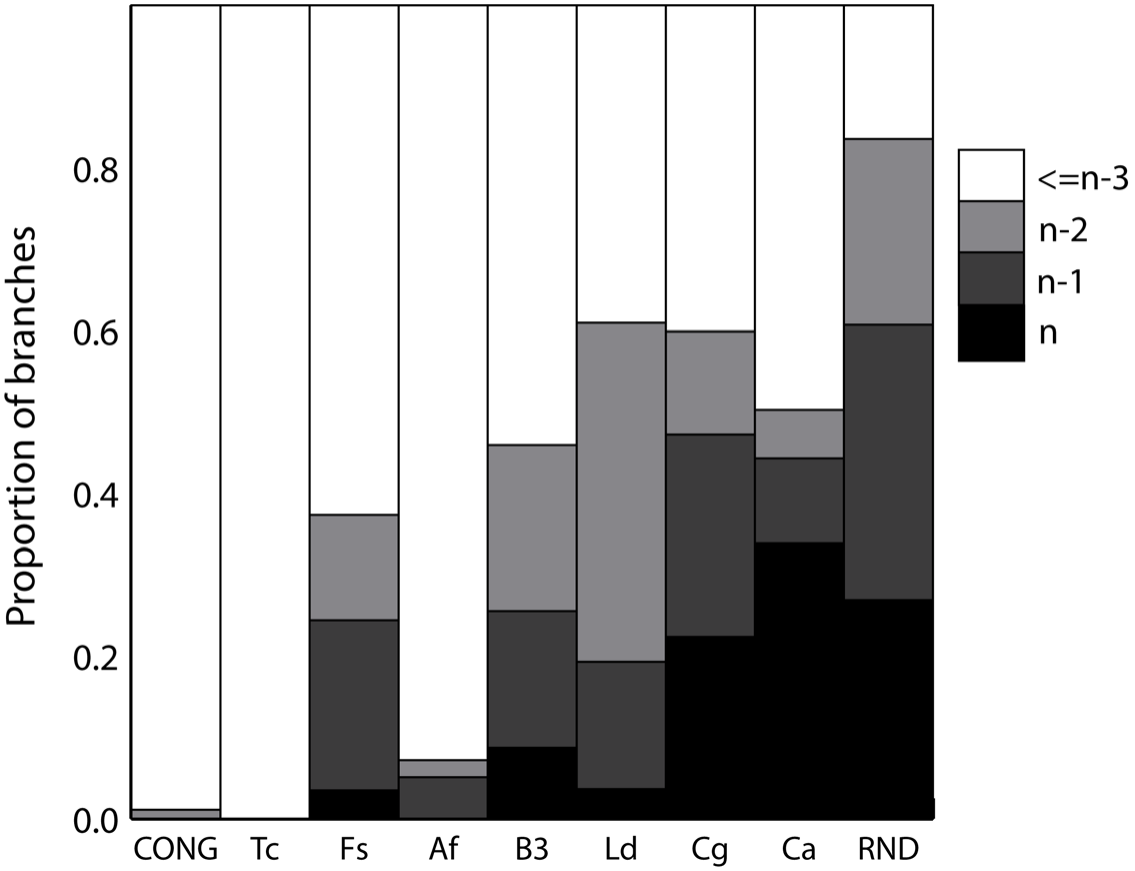
Topological incongruence distribution for standardized datasets. The color scale-bar represents the number of fragments topologically incompatible with certain branch. It varies from n incongruent fragments (black bars) to less than n-3 (white bars), where n is the number of fragments of the dataset. The values are calculated as the mean of 10 replications.

Overall, high support for most branches is indicative of a strong genetic structure. However, low support suggests two possibilities: either high incongruence or low information level. The difference between both possibilities is that high levels of incongruence are an indication of a weak structure, whereas low level of information is still compatible with a strong structure. Consequently, we analyzed incongruence levels in order to discriminate between both possibilities. We first analyzed overall incongruence using the BIONJ-ILD test available in MLSTest. The incongruence test was highly significant for all datasets (p value <0.01, 100 permutations), with the exception of *T. cruzi*. The last dataset has non-significant BIONJ-ILD (p = 0.31, 100 permutations). Moreover, BIONJ-ILD was still significant in subsamples of 24 STs (p<10^−4^ for *C. albicans*, *C. glabrata* and *A. fumigatus* and *F. solani* complex in all samples, p<10^−3^ for *L. donovani* complex). *Blastocystis spp* had variable p values for different subsamples (ranging from <10^−4^ to 0.068). We then analyzed how incongruence is distributed across the trees. Particularly, *C. albicans* and *C. glabrata* showed high levels of Topological Incongruence (TI) (Figure 3 and Figure S4). Both datasets showed more than 40% of the branches with a TI ≥ n-1 (where n is the number of fragments of the dataset). *C. albicans* and *C. glabrata* also had a mean TI ≥4 fragment per branch, whereas the mean TI was <4 in *T. cruzi*, *A. fumigatus*, *L. donovani* complex, *Blastocystis spp* and *F. solani complex* (Figure S4). This level of incongruence supports the hypothesis of weak structuration for *C. albicans* and *C. glabrata* datasets. Interestingly, *Blastocystis spp*, *A. fumigatus* and *L. donovani* complex, although they had low branch support, exhibited low to moderate levels of TI (between 5% and 25.5% of branches with at least n-1 incongruent fragments). Similar results were obtained using subsamples of the full set of strains for each micropathogen instead of the set of STs (Figure S3B). These results suggest that the observed low support for branches in *A. fumigatus*, *Blastocystis spp* and *L. donovani* complex implies a low number of polymorphic sites rather than a high level of incongruence.

**Figure 4:**
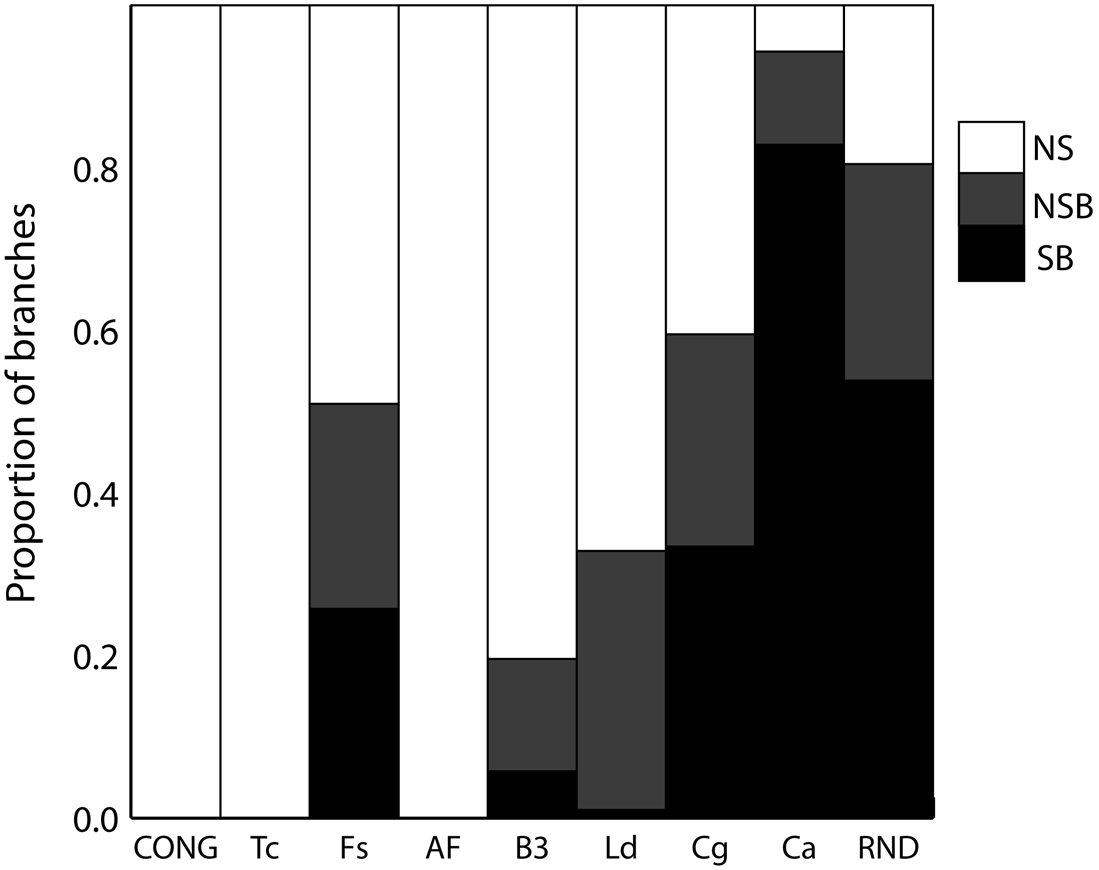
Significance distribution of NJ-LILD test for standardized datasets. The color scale-bar represents the p-value significance level for the test. NS, not significant at alpha = 0.05; NSB, not significant after Bonferroni correction; SB, significant after Bonferroni correction.

Topological incongruence is still possible in datasets having evolved under congruence (i.e. just by homoplasy, see the CONG dataset in Figure 3). Therefore, we analyzed the statistical significance of the topological incongruence using the NJ-LILD test (Figure 4). Again, *C. glabrata* and *C. albicans* showed 33% and 82.8% of the branches with significant localized incongruence (after Bonferroni correction), respectively. These results favor a weak structuration of these two datasets relative to the others. Other datasets showed either none or fewer than 30% of the branches with significant incongruence after Bonferroni correction (Figure 3). TI for CONG dataset was not significant by NJ-LILD test at any branch, as expected.

### 3.4 Genetic structure in *C. albicans*

We particularly analyzed the *C. albicans* dataset because our results are in apparent contradiction with previous results that strongly suggest PCE and discrete clusters [3], [30]–[33], based on the agreement between various different markers (congruence principle [34]). First, we analyzed a reduced dataset of 18 strains previously identified belonging to the near-clades I, II, III and SA by different methods (MLST, Ca3 fingerprinting, and microsatellite). The tree for concatenated sequences showed the four near-clades with considerable bootstrap support (Figure S5 upper branch values), an indicative of genetic structure. We also observed low topological incongruence (ranging from 3 to 4 loci per branch) in the previously described near-clades (Figure S5). Significant incongruence was observed for three main near-clades (p values <0.003, Figure S5). However, congruence among distance matrices of different fragments was statistically significant for this dataset and for a dataset of 60 randomly selected STs (Table 2). These results suggest that although incongruences are present, they are not sufficient to disrupt the genetic structure of the main near-clades.

**Table 2:** Congruence among distance matrices of different MLST fragments (AAT1, ACC, ADP, MPB, SYA, VPS, ZWF) for two different datasets of *Candida albicans*.

Null Hypothesis (H0)	Datasets	
	18 STs	60 STs
All matrices incongruent	0.0002*	0.0002
AAT1 incongruent	0.0128	0.0102
ACC incongruent	0.0002	0.0004
ADP incongruent	0.0002	0.0002
MPB incongruent	0.0002	0.0002
SYA incongruent	0.0546	0.0008
VPS incongruent	0.0002	0.0002
ZWF incongruent	0.0002	0.0002

*p value calculated from 5000 permutations.

**Figure 5:**
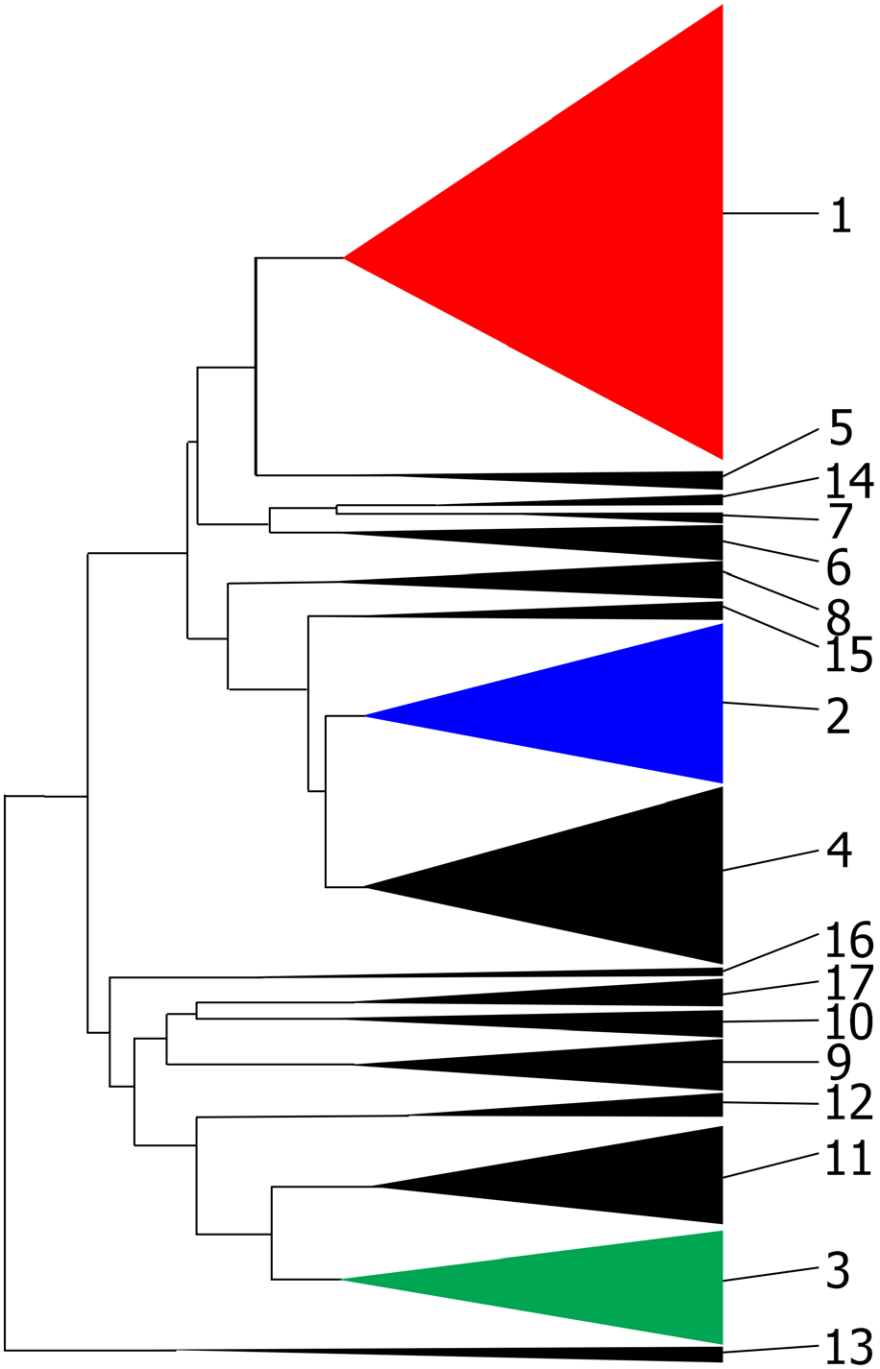
MLST clade architecture proposed by [28] for *Candida albicans*. Singleton STs are excluded and only the clades are shown. MLST clade 1 (red), MLST clade 2 (blue) and MLST clade 3 (green) were analyzed in the present work.

Odds et al. [28] proposed the existence of clades based on MLST for the 1,391 strains of *C. albicans* based on a dendrogram of concatenated fragments (Figure 5). The criterion to subdivision was an arbitrary distance cut-off. We analyzed whether three of these putative near-clades (particularly near-clades 1 to 3, See colored clades in Figure 5) were reliable or artifactual in the 60 STs dataset. First, we observed that MLST clades 2 and 3 do not formed a monophyletic group in the tree of the concatenated dataset (Figure S6). Moreover, they were not groups in any of the individual fragments (Figure S6). We further analyzed whether concatenated datasets supported clusters, even though monophyly was not fulfilled. This was made using a Mantel test to compare correspondence between the matrix of concatenated fragments and a model matrix which differentiate just one of the three clades. We observed that the matrix of concatenated fragments has no significant correspondence with matrices that differentiate MLST clades 2 or 3 (Table 3). This is important because, although there is general congruence among matrices, the clusters previously observed only by MLST could be artifacts of concatenation and would require further validation. Additionally, although the distance matrix of concatenated fragments had correspondence with the clade 1 matrix, just 2 out of 7 individual fragments had significant correspondence in the Mantel test (Table 3). This may be caused by artifacts of concatenation. However, we cannot discard that it may be caused by real but non-significant congruence with clade 1 for MLST fragments, the congruence being only detected after concatenating. This is an illustration of the congruence principle [34].

**Table 3:** Significance of different near-clades in distance matrices of the 60 STs dataset of *Candida albicans*.

		Locus						
Near-Clade	Concat^1^	AAT	ACC	ADP	MPB	SYA	VPS	ZWF
1	**0.0002** 2	3.27	0.06^3^	0.45	0.08^3^	**0.0014**	1.80	**0.0014**
2	0.86	0.15^3^	1.61	6.14	6.40	0.18^3^	3.55	5.96
3	0.26	6.75	1.31	**0.0028**	**0.014**	6.32	0.98	1.86

1 Distance matrix for concatenated dataset.

2 Bonferroni corrected p value for Mantel test with 5,000 random permutation. A significant value (p<0.05) means congruence between the distance matrix and a binary distance matrix that discriminate just one of the proposed near-clades.

3 Significant p values before Bonferroni correction.

### 3.5 Limitations of MLST to infer clades in congruent datasets

Finally, we analyzed whether MLST is useful to define near-clades in congruent datasets of moderate size. We used simulated congruent datasets of 68 taxa based on the tree for concatenated dataset of *C. glabrata* (Figure S7). Assuming that the tree displays the true evolutionary history for the species and that populations are strictly clonal, we evaluated the percentage of wrong clusters for datasets of 7 loci in 50 replications. The mean percentage of wrong clustering was 31±4.2%. Some branches from the true tree were recovered in less than 50% of the phylogenetic inferences (Figure S7). Interestingly, these branches were short branches (Figure S7). Many affected branches were deep branches and this is of importance because near-clades are frequently defined at deep branches. Additionally, if we used 10 or 20 fragments per scheme instead of 7, the level of wrong clustering was reduced to 18.9±2.8% and 12.9±1.5%, respectively. These results suggest that simulated MLST schemes of 7 loci had not enough polymorphic sites to resolve some deep branches in moderate to large datasets. The concatenated trees for each simulated dataset showed topological incongruence ranging from 0 (no incongruence) in branches close to leaves and 7 (maximum) for certain deep branches (Figure S8). However, the BIONJ-ILD p value was not significant in any branch, as it is expected. These results suggest that deep MLST clades obtained by concatenating should be carefully considered especially if they are defined by short branches.

## Discussion

Multilocus Sequence Typing allows comparative population structure analyses among pathogens. We have compared the degree of genetic structure of several online MLST datasets of eukaryotic microbes. Here, the genetic structure of the datasets under survey was analyzed considering the level of branch support and the level of incongruence. We implemented different measures of incongruence. Particularly, we used tests with different null hypotheses such as ILD-tests and CADM. Although other congruence tests between trees are available, such as the Congruence index (Ic) [35] based on the size of the maximum agreement subtree (MAST), they are not suitable for MLST data. This is because MLST fragment trees are usually partially-resolved (non-binary) trees, while this test is designed to compare fully-resolved trees. The obtained results strongly suggest variable levels of clonality ( = restrained recombination) and a notable evolutionary impact of genetic exchange in several of the species under study.

### 4.1 Genetic structure

Three different genetic structure types (GST) may be proposed according to the analyzed data (Table 4):

**Table 4:** Summary of dataset structure types found in our comparative analyses.

	Structure type		
	1	2	3
Consensus support^1^ *	Moderate to high	low	low
Bootstrap^2^ *	Moderate to high	low	low
BIONJ-ILD pval	variable	<0.01	<0.01
Topological incongruence^3^ *	Low to moderate	Low to moderate	High
Branches with significant LILD^4^	few	Few	More than 40%
Datasets	*T. cruzi,* *F. solani complex*	*A. fumigatus*, *Blastocystis* subtype 3,*L. donovani* complex	*C. albicans, C. glabra*ta

1 Consensus support was arbitrarily considered moderate for datasets with 30–60% of branches supported by at least two fragments and High for datasets with more than 60%.

2 Bootstrap support was arbitrarily considered moderate for datasets with 40%–60% of branches supported by bootstrap higher than 80% and High for datasets with more than 60% with bootrstrap value higher than 80%.

3 Topological incongruence was considered moderate for datasets with 20–40% of branches with n-1 fragments topologically incompatible with the validity of the near-clade in the concatenated tree and high incongruence was considered for datasets with more than 40% of branches with n-1 fragments topologically incompatible.

4 Significant NJ-LILD after Bonferroni correction.

*Thresholds are only used to define limits to different genetic structure types, which clearly emerge from a visual comparison of Figures 1, 2 and 3, S2, S3 and S4. It is important to note that the used thresholds are applicable to define structure types when only datasets around 24 STs are used.

GST 1: datasets with moderate to well-supported branches and relatively low levels of incongruence. They may be considered as the best-structured datasets. These include *T. cruzi* and *F. solani* complex. This degree of structuration is explained by PCE. This should be the case for *T. cruzi* [1]–[5], [36]. Our results are in agreement with another MLST scheme for *T. cruzi* [37]. These authors observed a fair branch support and a low level of incongruence that were caused by a few loci only. However, as recalled many times in the framework of the PCE model [1]–[5], [36], these results do not rule out the possibility of occasional genetic exchange in this pathogen. Genetic exchange has been demonstrated in laboratory experiments [38] and it is known that at least two of the six main *T. cruzi* near-clades have a hybrid origin [39]–[42]. Our results are in favor of very limited genetic recombination among *T. cruzi* near-clades. We also observed low levels of incongruence and moderate support within the near-clade TcI from a restricted geographical area (data not shown) suggesting low frequency of genetic recombination. This suggests that PCE operates within this near-clade too, according to the “Russian doll model”: within each near-clade, one observes a miniature picture of the whole species, with linkage disequilibrium and lesser near-clades [4].

The *F. solani* complex dataset was clearly attributed to GST 1 too. This is in agreement with another multilocus analysis with high support for branches [43]. However, *F. solani* is a complex of species [44] and our results show that within the complex, recombination is restrained. However it is impossible to know whether the complex as a whole undergoes PCE, or whether recombination is restrained among species, and not within them. Additional analyses will need to be performed within each of the subdivisions uncovered by our analysis, to ascertain whether these subdivisions correspond to near-clades generated by PCE (“Russian doll model” [4]), or to biological species within which recombination occurs at random. A sexual cycle has been evidenced in several species of the *F. solani* complex [44], [45]. However, the frequency of genetic exchange in natural populations of this fungus is unknown.

GST 2 corresponds to datasets with weakly-supported branches, but with low levels of incongruence. These include *A. fumigatus*, *L. donovani* complex and *Blastocystis* ST3. The level of genetic structuration of the dataset is therefore not clearly defined by MLST. There are two possible explanations for this structure type. First, it may obtain if the dataset corresponds to a population of recent origin having undergone a radiation process. Although major clusters can be identified in the *Blastocystis spp* dataset, the majority of the STs corresponding to SSU clade 1 [19] appear to fit a radiation structure pattern because this clade shows multiple polytomies.

The second explanation for the GST 2 is that the markers used for typing have a low level of informative polymorphisms. The *A. fumigatus* dataset also falls into this structure type. Although it has been proposed that *A. fumigatus* exhibits two clusters by MLST [18], these clusters have a low level of support in our analyses. Low levels of variability were observed in our dataset and for other markers [46]. This low level of variability is probably the cause of non-significant linkage disequilibrium (type II error) observed in many population studies [47], [48]. The only one study using a high number of individuals and a highly polymorphic marker (microsatellites) showed linkage disequilibrium in 4 out of 5 populations [49]. Consequently, *A. fumigatus* will require very polymorphic markers in order to analyze in depth its genetic structure and to determine the impact of its sexual cycle in natural populations.

The *L. donovani complex* dataset is referred to as GST 2. A low number of polymorphic sites was observed for the dataset despite the fact that long fragments (more than 1,000 bp) were analyzed. For example, quite low support was observed for the group *L. infantum*. However, other markers strongly suggest that the group is monophyletic [50]. PCE and “Russian doll” has been proposed for *Leishmania spp*. [3]–[5]. Unfortunately, the dataset used here does not give information about the role of genetic exchange.

GST 3: weakly-supported branches and high and significant levels of topological incongruence. They should be considered as weakly-structured datasets. *Candida* datasets exhibit this pattern. However, not all weakly-structured datasets by MLST imply that the species under survey is poorly structured. At least 5 near-clades were evidenced in *C. albicans* with strong (although not total) correspondence between different markers [30], [32], [33], [51]–[53] which corresponds to the congruence criterion of PCE [3], [34]. The Mantel correlation test has shown a highly significant correlation among markers in *C. albicans* [51], [52], which again strongly supports the PCE congruence criterion. We observed the same for our datasets, where significant congruence was observed among Distance matrices for different MLST fragments, which also means linkage disequilibrium. However, incongruence was still significant. So, the relatively weak structure pattern for MLST datasets proposed here is not against the existence of near-clades. However, our results suggest two important things about the *C. albicans* MLST dataset: a role of recombination in blurring the *C. albicans* genetic structure and certain limitations of MLST used alone to define clusters. Odds et al. [28] also observed incongruence among trees in a multilocus phylogenetic analysis of *C. albicans*. Recombination may cloud the structure of the species in MLST datasets, because some markers (such as MLST targets) could be more “sensitive” to the “clouding impact” of genetic exchange. This clouding is understood here as the effect of occasional recombination among strains or group of strains, which impedes that trees show well-supported clades by a little blurring of the limits between them. Although there are explanations for incongruence in diploids other than genetic exchange, they should not have sufficient impact. For example, events of Loss of Heterozygosity (LOH) may produce incongruence. However, similar levels of incongruence are observed in the relative *C. glabrata* dataset where LOH is not expected because is mainly haploid, suggesting that this mechanism is not the only cause of incongruence in *C. albicans*. The second important finding is that MLST could lack sufficient resolution to resolve certain branches in large datasets (particularly important for deep branches). Therefore, the cloud effect in *Candida albicans* MLST could be a combination of both factors, sensitiveness of MLST analyses to low levels of genetic exchange and lack of resolution to resolve certain near-clades.

While it is true that no sexual cycle is known for *C. glabrata*, it is clear that genetic exchange has had an impact in the evolutionary story of the species. Evidence of recombination has been shown for *C. glabrata* in 165 isolates among which 14 phylogenetic incompatibilities were found [54]. Another *Candida* dataset (*C. tropicalis*) available online showed a very similar structure to *C. albicans* (data not shown) suggesting that weak structuring by MLST is characteristic of the genus. Altogether, our results are compatible with the PCE model in *Candida*. However they bring additional information to it, which makes it possible to propose that genetic exchange was rare but enough to generate a blurring in particular regions of the tree-like population structure of this yeast.

### 4.2 About what GSTs are saying

It is tentative to compare with the same tools radically different species, because this implies to operate at different scales; that is to say, different sampling strategies, different evolutionary times and different variability levels. Our tests deal with datasets, not species, and should be extrapolated cautiously to whole species. In this sense, scale is a crucial factor when whole species rather than only datasets are considered. This can be illustrated by the following example. If we analyze a mammalian dataset (for example, several apes, artyodactils, canids and felids), we will conclude that it is a well-structured dataset, which is right (well-supported branches and low incongruence). This result obviously is not due to PCE. However, the conclusions about the dataset are important in themselves because they will orientate the following analyses and generate questions about the organism. Lastly, other GSTs, apart from the ones exposed here, are possible. For example, datasets with overall significant bootstrap but high incongruence have been observed in *Neisseria meningitidis* [25].

### 4.3 Delimitation and validation of near-clades based on MLST data

It is clear that near-clades, with the definition proposed by Tibayrenc and Ayala [3], should be cautiously considered in the case of GSTs type 3 because tree-like methods may produce artifactual groups due to incongruent phylogenetic signals. Even in structured species (with defined near-clades), if the MLST dataset has a GST type 3, there is no guarantee that the observed clusters are reliable. It is necessary in this case to corroborate them by the use of other genetic markers [3]. We propose that the existence of near-clades, when the specific case of MLST data is considered alone, should be based on branchings with a fair support (bootstrap and consensus support), and a low level of incongruence. Additionally, clustering should be carefully considered in the case of small datasets; for example, when they include a limited number of STs. If these conditions are not fulfilled, other methods than MLST and concatenation are needed to corroborate the presence of near-clades.

### 4.4 Concluding remarks

The PCE model of pathogenic microorganisms, as defined by Tibayrenc and Ayala [3], [4], is of crucial interest, because it provides a convenient framework for all applied studies dealing with microbes, including molecular epidemiology, clinical studies, vaccine and drug design. In consequence, our ability to determine the population structure of pathogens is crucial. In the present study, we have exposed methods that could be helpful for identifying the level and reliability of genetic structuration of pathogenic microorganisms, and we have shown some limitations of the use of MLST data to identify near-clades in predominantly clonal microorganisms. Although MLST is being over-competed by whole genome sequencing (WGS) strategies in bacterial organisms, this typing method applied to large sample sizes is not currently viable because of its still high costs for eukaryotic genomes. Therefore, it would be expected that MLST will not be replaced by WGS in a close future. In addition, the structure types and related questions dealing with genetic structure analyzed here are still valid for WG analysis. Lastly, WGS approaches cannot be standardized the same way than MLST, since they involve radically dissimilar parts of the genome, with considerable differences among different types of pathogens.

## Supporting Information

Figure S1 Neighbor Joining tree for 1000 ST from *Candida albicans* using uncorrected p-distances. Branches are colored according to the Consensus Support (CS). Red branches have CS = 0. Orange branches have CS = 1. Yellow branches have CS = 2. Green branches have CS>2. Grey branches are terminal branches.
(TIF)

Figure S2 Standardized Mean Consensus Support for each dataset. The mean consensus support was standardized at 7 loci. The error bars represent the 95% confidence interval for the standardized mean.
(TIF)

Figure S3 Consensus support (A) and Topological Incongruence (B) distribution for datasets of 24 randomly selected strains. The values are calculated as the mean of 3 replications. See legends of Figure 1 and Figure 3 for further explanations of the color scale-bars in A and B, respectively.
(TIF)

Figure S4 Standardized Mean Topological Incongruence for each dataset. The mean Topological Incongruence was standardized at 7 loci. The error bars represent the 95% confidence interval for the standardized mean.
(TIF)

Figure S5 Neighbor Joining tree for *Candida albicans* dataset of 18 strains previously identified, belonging to the near-clades I, II, III and SA (vertical bars). Bootstrap values over 60% (upper branch values), topological incongruence (lower left branch values) and NJ-LILD p values for the near-clades (lower branch values) are shown.
(TIF)

Figure S6 Multiple topological incongruences in a random dataset of 60 STs of *Candida albicans*. The first tree is based on concatenated fragments (concatenated) and the following trees correspond to fragments trees (the fragment name is indicated at the top-left of each tree). Red boxes represent STs from MLST clade 1, blue boxes represent STs from MLST clade 2, and green boxes represent STs from MLST clade 3.
(TIF)

Figure S7 Percentage of wrong branches for 50 datasets simulated along the showed tree. Branches in red were observed in less than the 50% of the replications of tree inference based on NJ method. Branches in orange, less than the 75%. Branches in green, more than 75%.
(TIF)

Figure S8 Concatenated tree for a simulated dataset of 7 congruent fragments. Topological incongruence is shown above the branch. Only values higher than 3 are shown.
(TIF)
