# Supplementary figures and images for: How Often Do They Have Sex? A Comparative Analysis of the Population Structure of Seven Eukaryotic Microbial Pathogens
Source: PLoS One. 2014 Jul 23;9(7):e103131. doi: 10.1371/journal.pone.0103131 (PMC4108389; doi:10.1371/journal.pone.0103131)

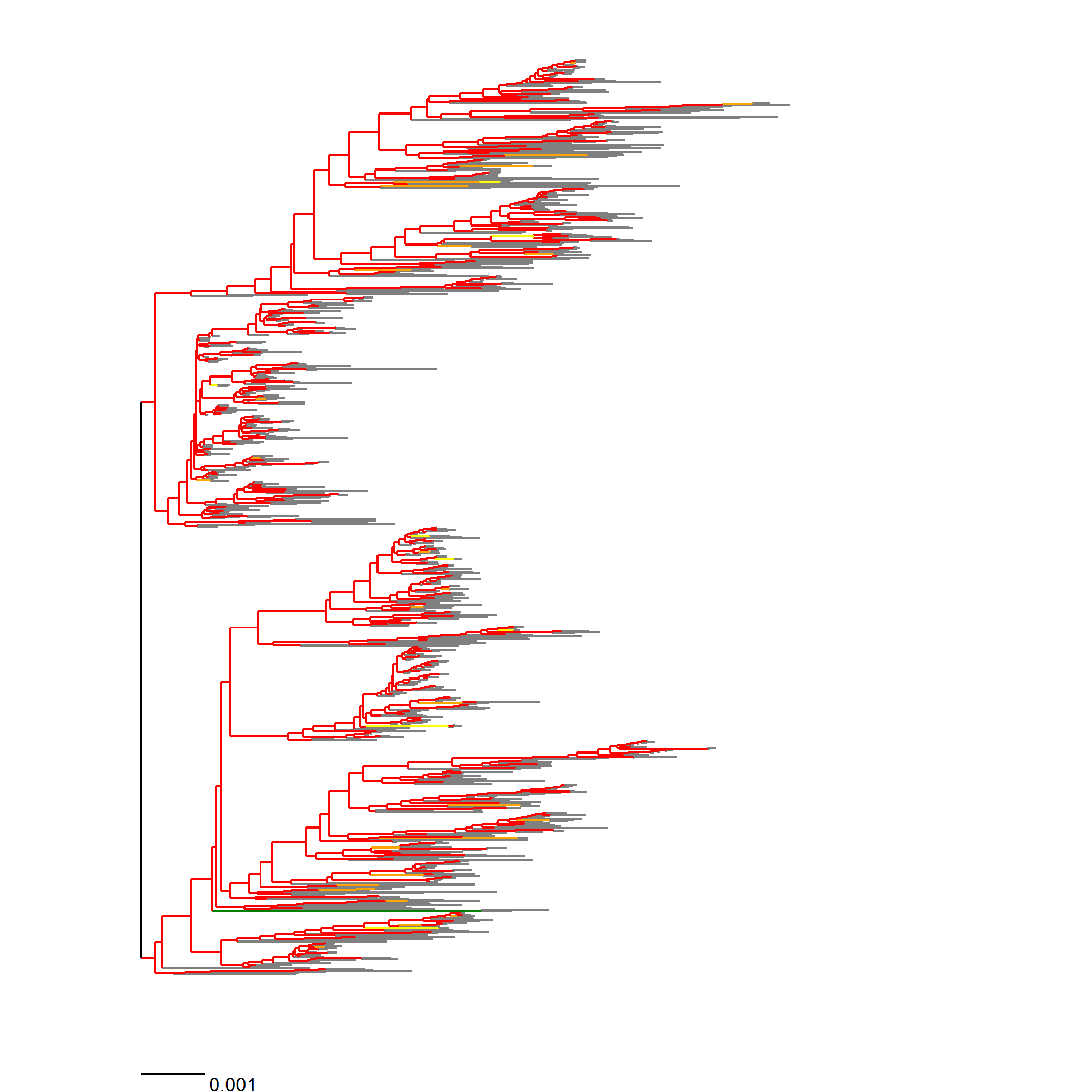

Supplement: Figure S1 — Neighbor Joining tree for 1000 ST from Candida albicans using uncorrected p-distances. Branches are colored according to the Consensus Support (CS). Red branches have CS = 0. Orange branches have CS = 1. Yellow branches have CS = 2. Green branches have CS>2. Grey branches are terminal branches. (TIF) [file pone.0103131.s001.tif]

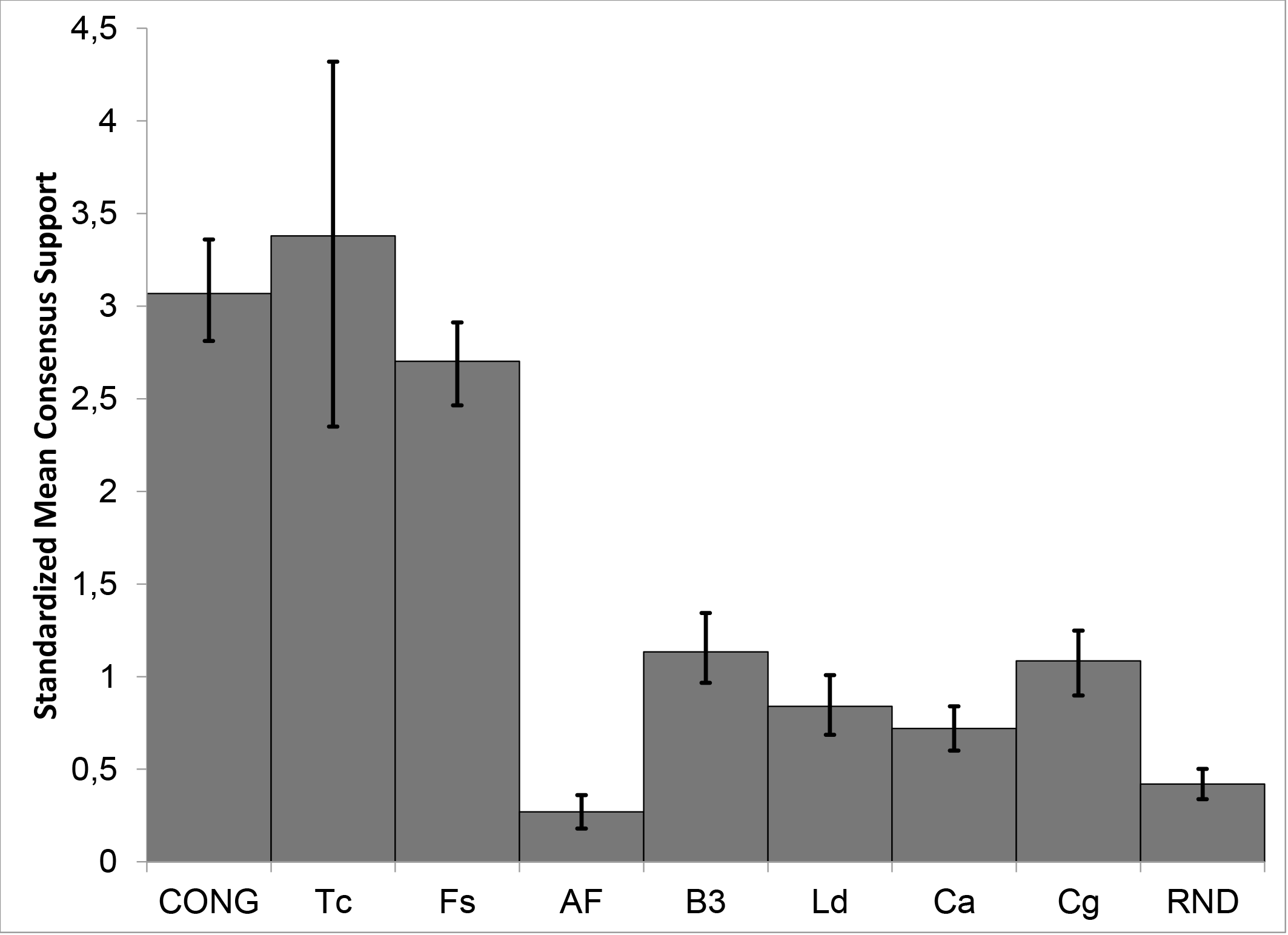

Supplement: Figure S2 — Standardized Mean Consensus Support for each dataset. The mean consensus support was standardized at 7 loci. The error bars represent the 95% confidence interval for the standardized mean. (TIF) [file pone.0103131.s002.tif]

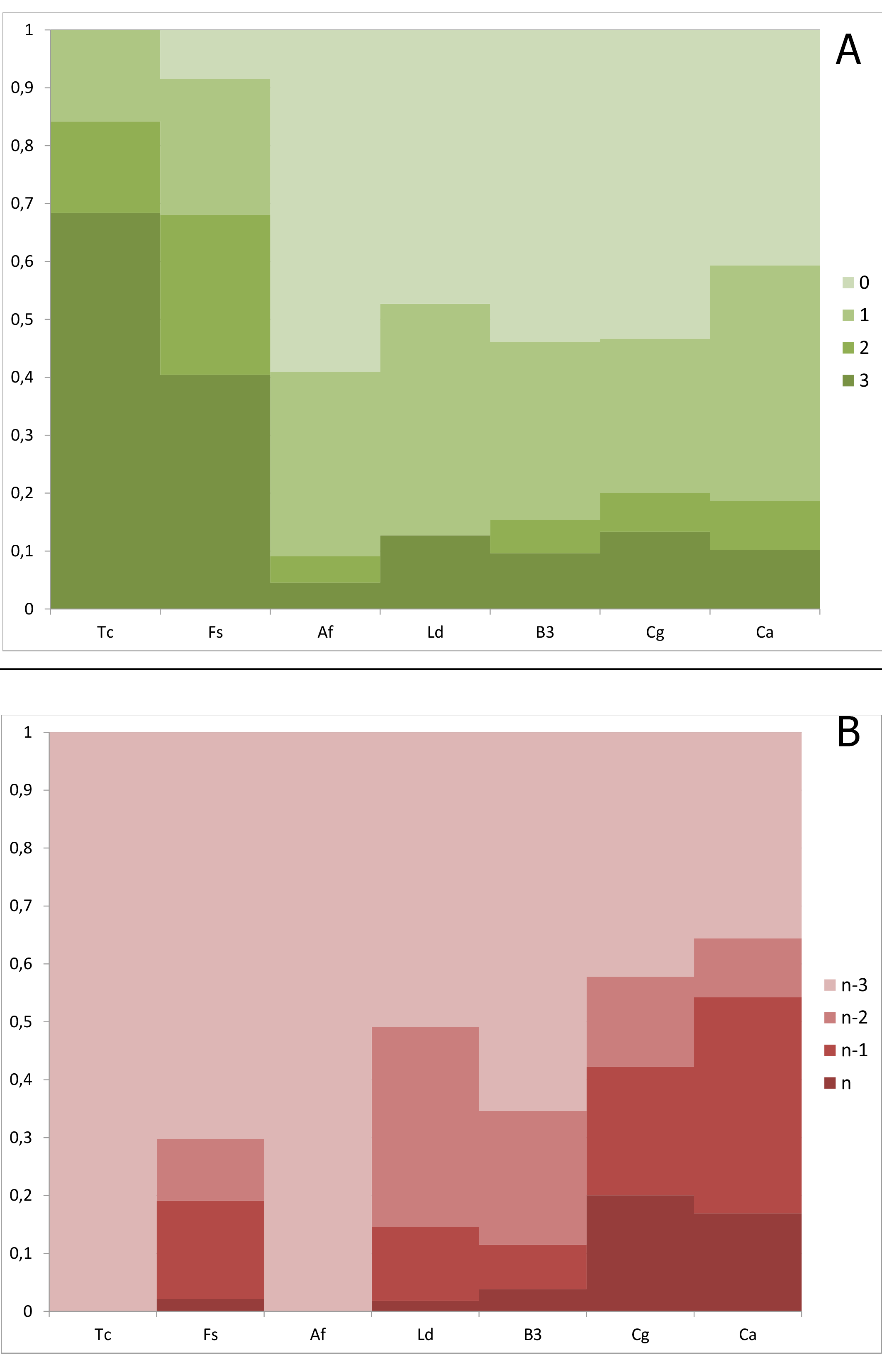

Supplement: Figure S3 — Consensus support (A) and Topological Incongruence (B) distribution for datasets of 24 randomly selected strains. The values are calculated as the mean of 3 replications. See legends of Figure 1 and Figure 3 for further explanations of the color scale-bars in A and B, respectively. (TIF) [file pone.0103131.s003.tif]

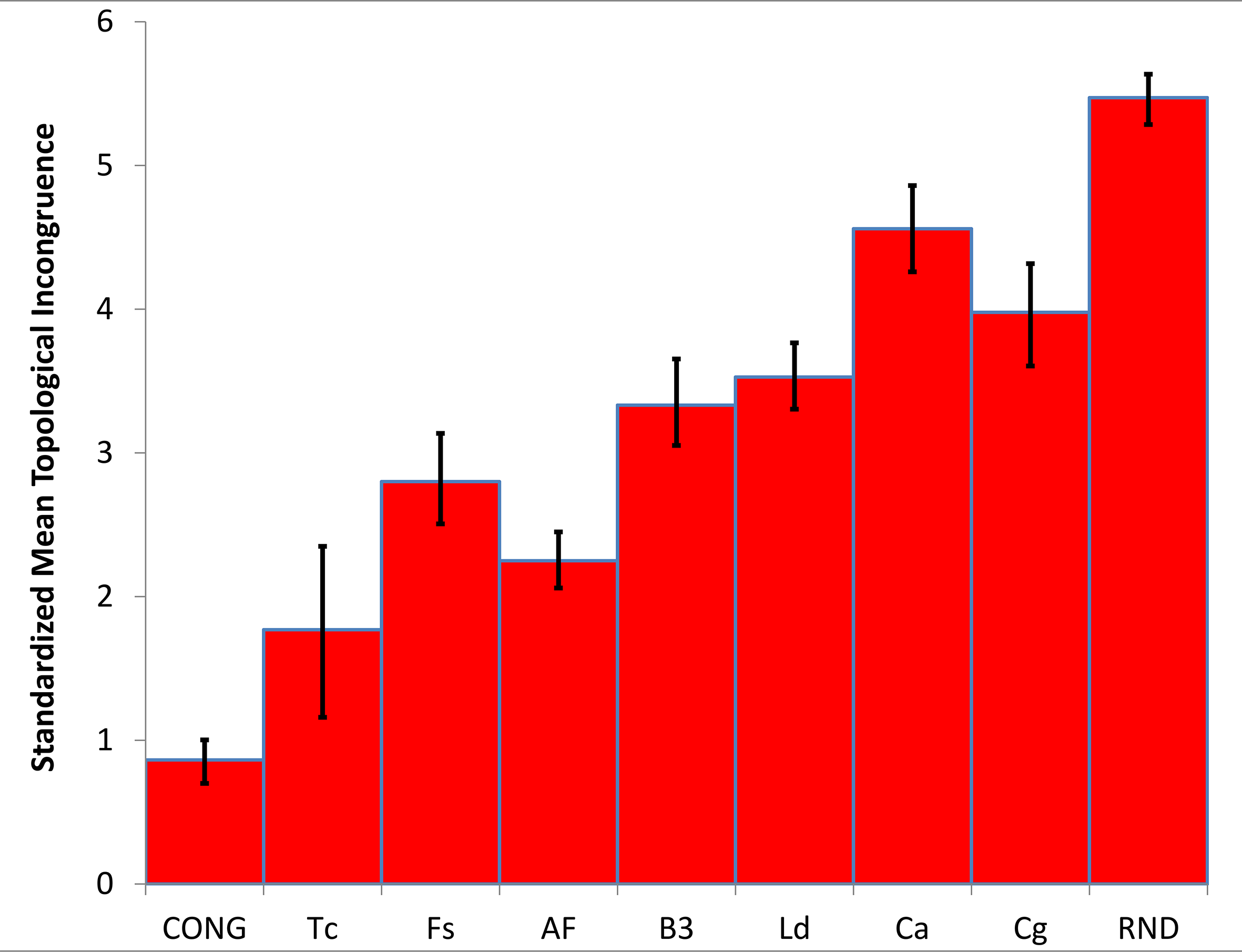

Supplement: Figure S4 — Standardized Mean Topological Incongruence for each dataset. The mean Topological Incongruence was standardized at 7 loci. The error bars represent the 95% confidence interval for the standardized mean. (TIF) [file pone.0103131.s004.tif]

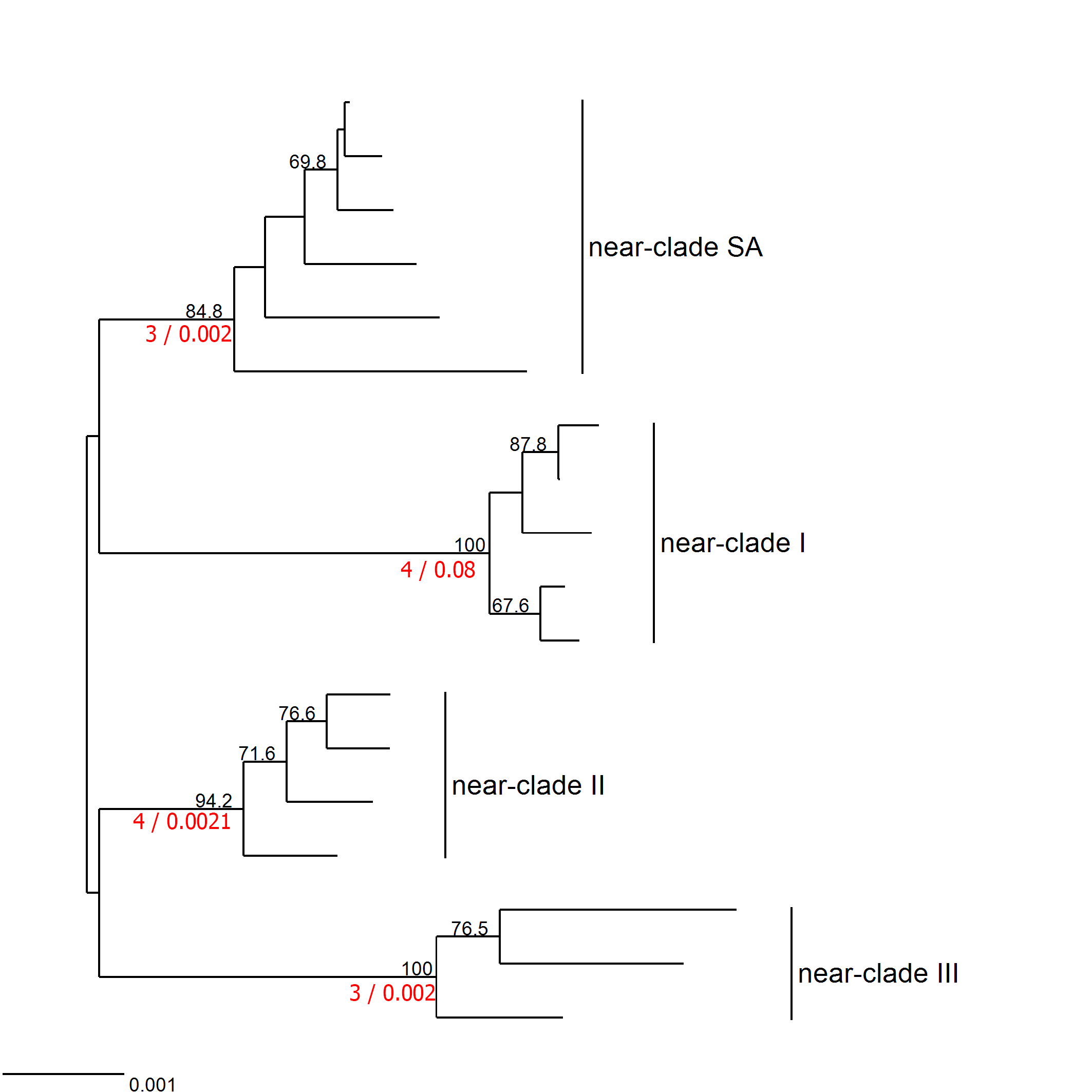

Supplement: Figure S5 — Neighbor Joining tree for Candida albicans dataset of 18 strains previously identified, belonging to the near-clades I, II, III and SA (vertical bars). Bootstrap values over 60% (upper branch values), topological incongruence (lower left branch values) and NJ-LILD p values for the near-clades (lower branch values) are shown. (TIF) [file pone.0103131.s005.tif]

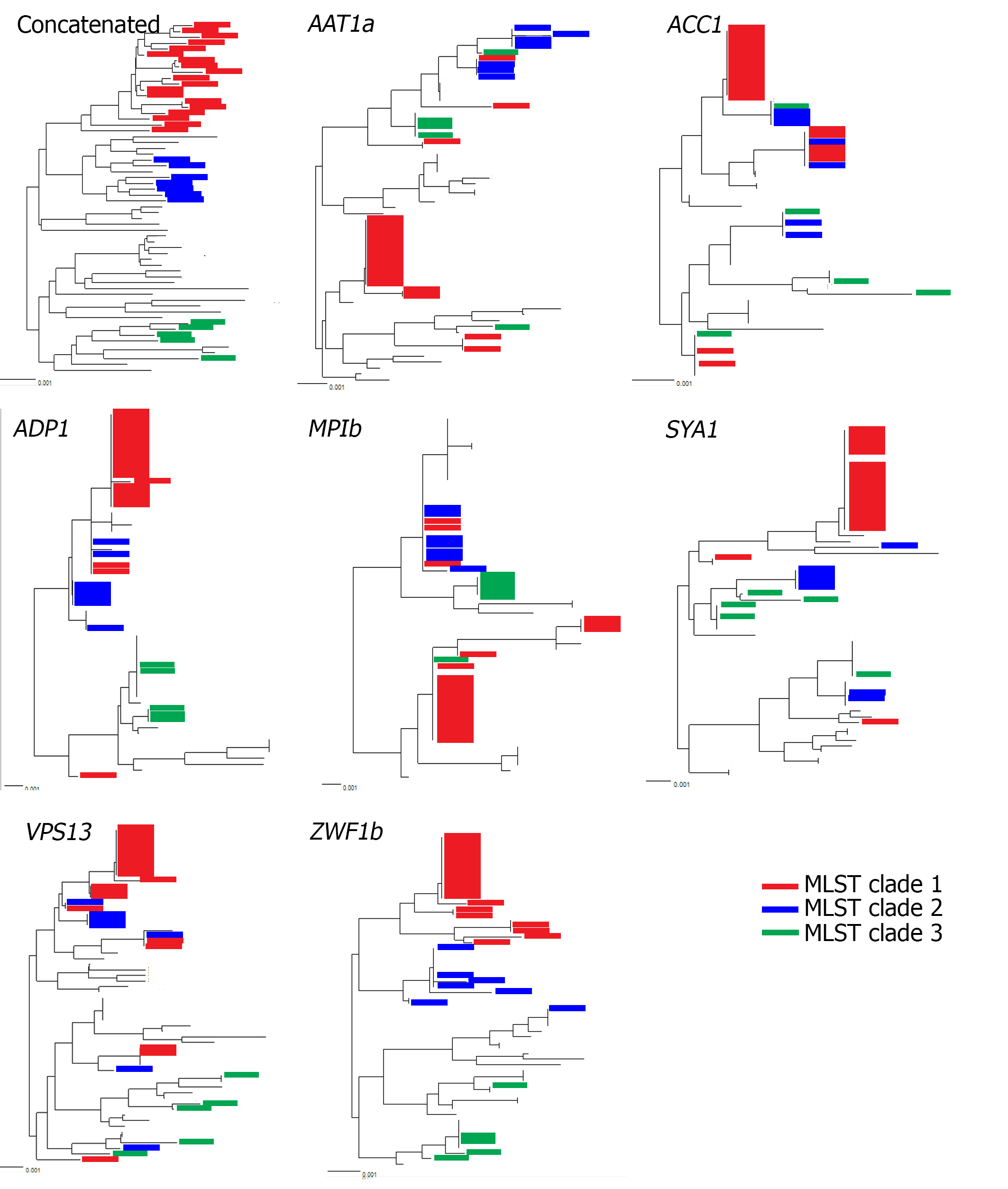

Supplement: Figure S6 — Multiple topological incongruences in a random dataset of 60 STs of Candida albicans. The first tree is based on concatenated fragments (concatenated) and the following trees correspond to fragments trees (the fragment name is indicated at the top-left of each tree). Red boxes represent STs from MLST clade 1, blue boxes represent STs from MLST clade 2, and green boxes represent STs from MLST clade 3. (TIF) [file pone.0103131.s006.tif]

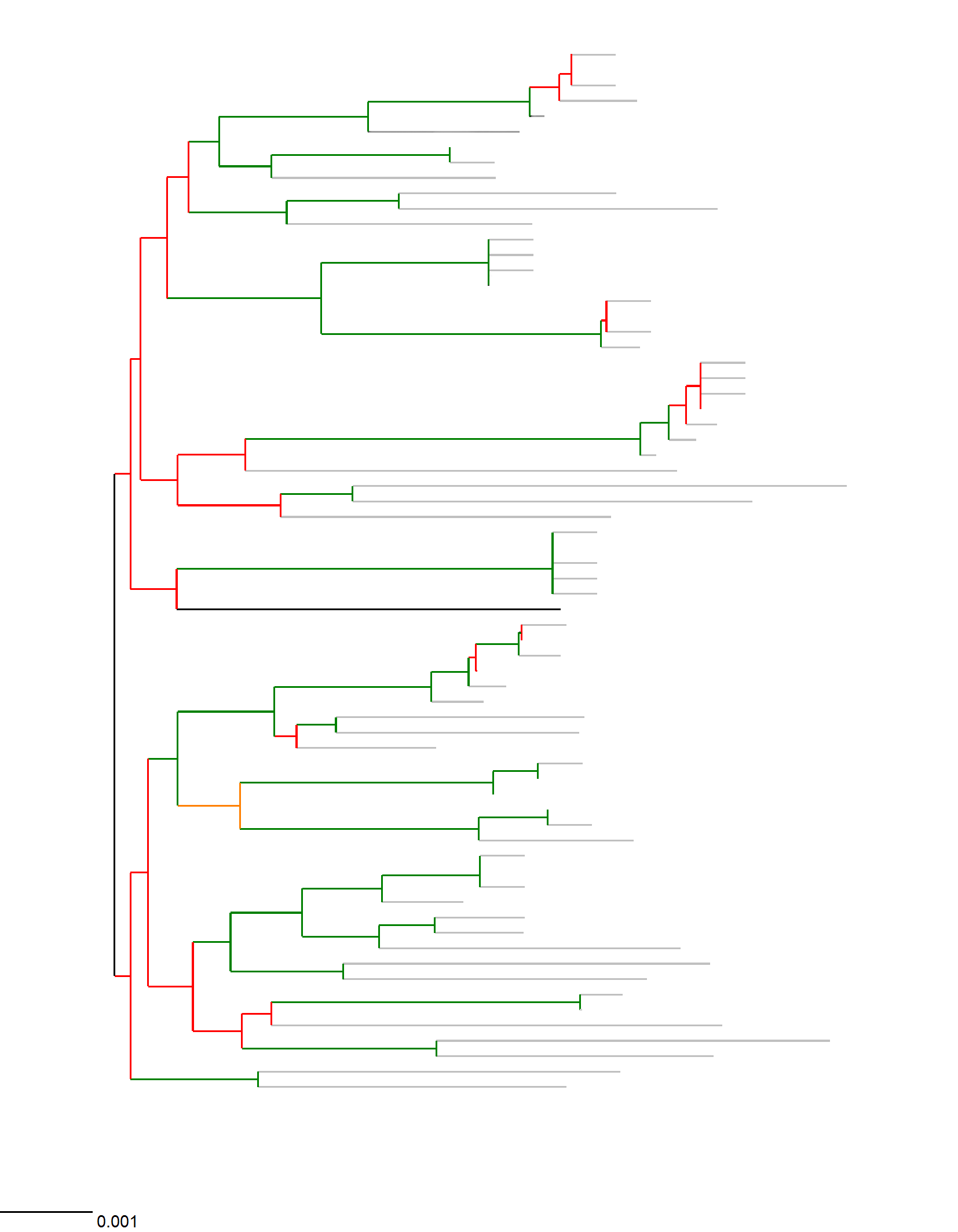

Supplement: Figure S7 — Percentage of wrong branches for 50 datasets simulated along the showed tree. Branches in red were observed in less than the 50% of the replications of tree inference based on NJ method. Branches in orange, less than the 75%. Branches in green, more than 75%. (TIF) [file pone.0103131.s007.tif]

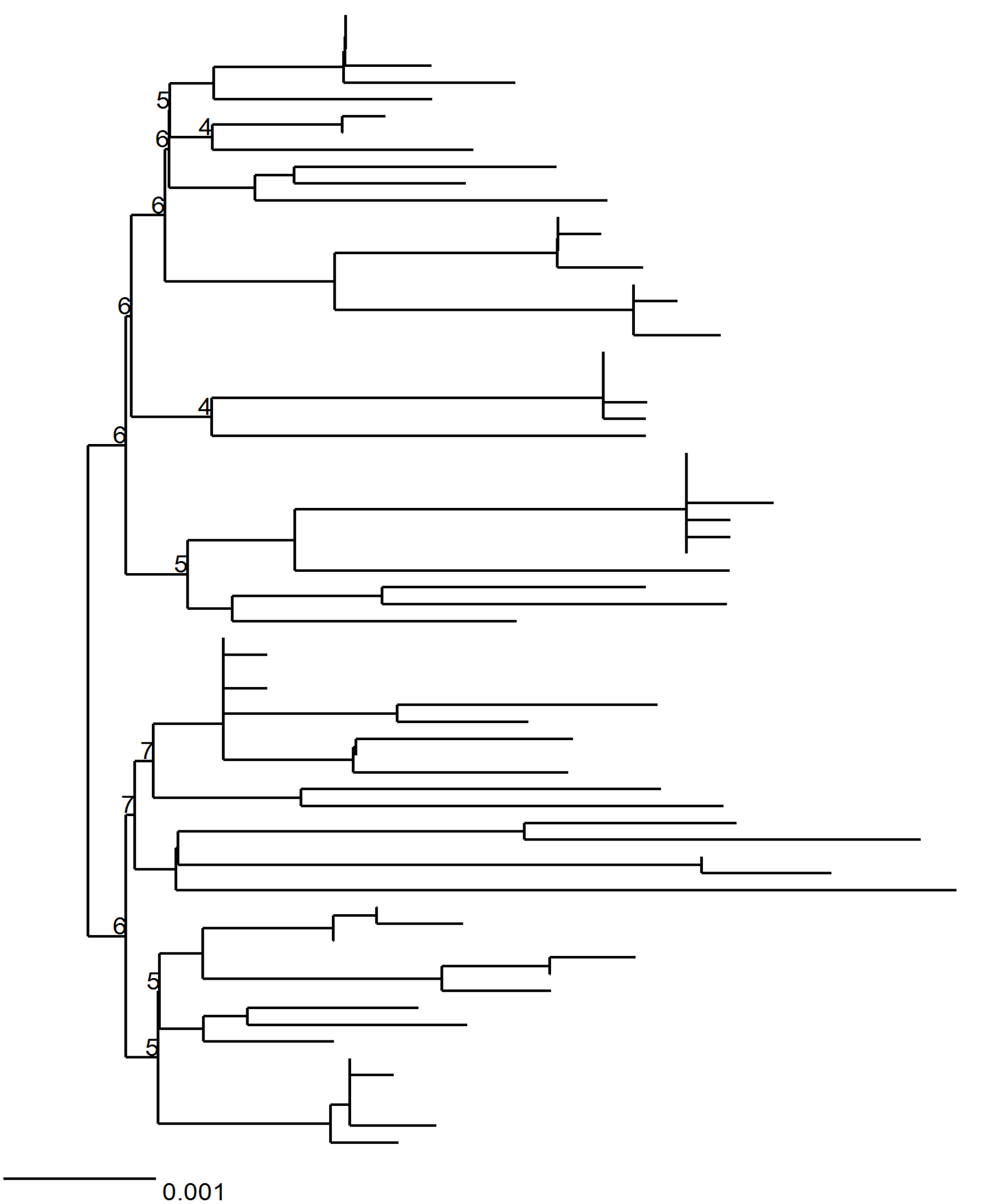

Supplement: Figure S8 — Concatenated tree for a simulated dataset of 7 congruent fragments. Topological incongruence is shown above the branch. Only values higher than 3 are shown. (TIF) [file pone.0103131.s008.tif]
